# Prognostic impact of lymph node count features in total laryngectomy for advanced laryngeal squamous cell carcinoma

**DOI:** 10.1007/s00432-025-06320-9

**Published:** 2025-10-07

**Authors:** Mihnea Cristian Trache, Lisa Budelmann, Philippe Christophe Breda, Jördis Kristin Eden, Stefan Bartels, Sophia Marie Häußler, Christian Stephan Betz, Jacob Friedrich Clausen, Lukas Wittig, Arne Böttcher

**Affiliations:** 1 Department of Otorhinolaryngology, University Medical Center Hamburg-Eppendorf, Martinistraße 52, 20251 Hamburg, Germany; 2 University Cancer Center Hamburg, University Medical Center Hamburg-Eppendorf, Hamburg, Germany

**Keywords:** Total laryngectomy, Neck dissection, Nodal yield, Lymph node burden, Lymph node ratio, LODDS

## Abstract

**Introduction:**

The optimal surgical management in advanced laryngeal squamous cell carcinoma (ALSCC) is still under debate. The extent of neck dissection as well as the nodal involvement affect survival metrics in head and neck cancer (HNSCC) patients. Despite pN status, other parameters like nodal yield (NY) or lymph node ratio (LNR) have been investigated before. There are data showing that log odds of positive lymph nodes (LODDS) are a good survival prognosticator in HNSCC in general but specific data on ALSCC is missing. This study aims to assess the prognostic value of lymph node count features on survival in ALSCC.

**Methods:**

We conducted a retrospective patient chart review on curative intent laryngectomy and bilateral neck dissection for ALSCC between 2009 and 2024 at a tertiary care center. Investigated lymph node count features besides NY included lymph node burden (LNB = number of positive lymph nodes), LNR (= LNB/NY) and the LODDS = log ((LNB + 0.5) / (NY-LNB + 0.5)). Univariate survival analysis was performed using the log-rank testing and Kaplan–Meier curves. The R maxstat package was utilized for an optimized cut-off point determination for cohort risk stratification.

**Results:**

We included 56 patients who underwent laryngectomy and bilateral neck dissection in our department. Survival analysis revealed a 5-year OS of 51% and median OS of 60.7 months. The LODDS ranged from -2.48 to 0.37 with a mean value of -1.68 ± 0.50. The cut-off at –1.94 for LODDS showed a 5-year DSF of 33% vs. 61% with a HR of 0.27 (*p* = 0.005) and the optimized cut-off of -1.55 showed significant differences in 5-year OS (69 vs. 17%, HR: 0.29, p = 0.003). LODDS indicated the highest concordance indices for both DFS and OS compared to LNB and LNR.

**Conclusion:**

We propose LODDS to serve as a superior prognosticator compared to LNB and LNR concerning DFS and OS in TL for ALSCC. LODDS values of -1.94 for DFS and -1.55 for OS appear as suitable thresholds for risk stratification.

## Introduction

Having experienced a distinct incidence decline within the last 30 years, laryngeal squamous cell carcinoma appears the 3rd most common head and neck cancer with an annual incidence of 3.200 cases in 2020 in Germany (Krebs in Deutschland für 2019). Surgery remains the major therapeutic option, especially in an advanced stage with cartilage involvement (cT4a) as it demonstrates a survival benefit compared to organ preservation protocols (Bootz 2020; Bozec et al. 2020).

The extent of regional lymph node involvement is a crucial factor when aiming to predict the patients’ outcome in surgically treated advanced stage laryngeal squamous cell carcinoma (ALSCC). This subentity of head and neck squamous cell carcinoma is distinct from others, e.g. oropharyngeal cancer, as it develops comparably late and sparse regional metastases (Böttcher et al. 2017). Furthermore, the pN status helps to assess the necessity for adjuvant therapy in each individual case (Bootz 2020). In recent years, several attempts were made in establishing better prognosticators than pN status alone such as nodal yield (NY) (Böttcher et al. 2016), lymph node burden (LNB) (Molteni et al. 2023), and lymph node ratio (LNR) (Molteni et al. 2023).

The pN status also highly depends on the extent of lymph node removal and loses validity in cases with poor NY. Therefore, parameters such as lymph node ratio (LNR = number of positive nodes / NY) and log odds of positive lymph nodes (LODDS = log ((LNB + 0.5) / (NY-LNB + 0.5))), which take NY into account, have been proposed and already established as prognostic markers in gastrointestinal cancer (Arslan et al. 2014; Qiu et al. 2012). In squamous cell carcinoma of the head and neck (HNSCC), both LODDS and LNR have repeatedly shown to correlate with overall survival, but LODDS might be a superior predictor (Kouka et al. 2022; Safi et al. 2017; Jin et al. 2020; Yildiz et al. 2016). LODDS especially is able to differentiate prognosis of patients with a low NY and achieve better prediction of survival than the pN status in oral cancer patients (Lee et al. 2017).

This study aims to evaluate the prognostic performance of regional lymph node count features in ALSCC.

## Methods

### Ethical statement

This article does not contain any experimental study with human participants performed by any of the authors. No identifying information is included in this article. Written informed consent was obtained from all individuals before surgical intervention. For this type of work, formal consent is not required due to its retrospective nature, according to § 12 HmbKHG (Hamburg hospital law).

Following institutional approval by the Clinical Cancer Registry of the University Cancer Center Hamburg, data were reviewed from all patients with histologically confirmed ALSCC who underwent primary total laryngectomy (TL) with bilateral neck dissection for curative intent at the University Medical Center Hamburg-Eppendorf between 2009 and 2024. Using specially trained coordinators, data were obtained from a database management system using GTDS (Gießener Tumordokumentationssystem; https:// www.med.uni-giessen.de/akkk/gtds/), which thoroughly documents patients’ features using the original pathology reports. Additionally, a review of patients’ digital records was conducted using our local documentation systems Torin OR Management (Getinge) and Soarian® Clinicals (Cerner). Cases were identified using the German Operation and Procedure Classifcation System (Operationen- und Prozedurenschlüssel, OPS) code 5-303 and the German modifcation of the International Classifcation of Disease (ICD-10-GM) for Oncology topography code C32.-. Each patient has been directly treated or examined on a follow-up routine by at least one of the authors. There were no cases excluded due to missing data.

Nodal yield (NY) was calculated as the number of bilaterally harvested lymph nodes. Lymph node burden (LNB) was defined as the total number of positive lymph nodes. Lymph node ratio (LNR) was calculated as the quotient of LNB divided by NY and the log odds of positive lymph nodes (LODDS) were calculated as log_10_ ((LNB + 0.5) / (NY-LNB + 0.5)).

Exclusion criteria included:

- salvage TL,
- TL after induction chemotherapy,
- TL due to hypopharyngeal SCC,
- functional TL,
- TL for non-SCC tumors,
- history of chemotherapy/-radiation,
- history of neck dissection,
- history of cordectomy > Type I, and
- multi-level growth.

### Statistical analysis

Statistical analysis was performed using Microsoft Excel 2021, as well as IBM SPSS Statistics (Version 29.0.1.0 (171)), and R (v4.4.2.; The R Foundation, https://www.r-project.org/). Statistical significance was set at a level of α = 0.05 (*p* < 0.05). Differences in survival were calculated from the date of TL to the date of death or last known follow-up (overall survival, OS) or to the date of first disease recurrence or death from any cause (disease-free survival, DFS). Differences in survival were analyzed using univariate regression analysis (generalized Wilcoxon Mantel–Cox log-rank for long-term follow-up) using the Chi-squared (χ2) statistic. Survival curves were generated using the Kaplan–Meier method. To determine the optimal cut-off point for one or multiple continuous variables at once, the maximally selected rank statistics the ‘maxstat’ R package is used. This is an outcome-oriented method providing a value of a cut point that corresponds to the most significant relation with survival outcome (Hothorn and Lausen 2003).

## Results

We identified 56 patients with a mean age of 65.5 years who underwent TL with bilateral neck dissection with curative intent. Of these, 98.2% presented with an advanced stage and 66.1% of the cases had to undergo adjuvant treatment (Table 1).

**Table 1:** Patient characteristics (n = 56)

Feature		N (%)
Age at TL	Mean = 65.5 years	
	< 65 years	27 (48.2)
	≥ 65 years	29 (52.8)
Sex	Male	52 (92.9)
	Female	4 (7.1)
pT^a^	2	1 (1.8)
	3	26 (46.4)
	4a	29 (52.8)
pN^a^	0	34 (60.7)
	1	4 (7.1)
	2a	0 (0.0)
	2b	4 (7.1)
	2c	4 (7.1)
	3a	0 (0.0)
	3b	10 (17.9)
ENE	Pos	10 (17.9)
	Neg	46 (82.1)
AJCC stage^a^	II	1 (1.8)
	III	20 (35.7)
	IVA	25 (44.6)
	IVB	10 (17.9)
R status	0	52 (92.9)
	1	4 (7.1)
Adjuvant treatment	None	17 (30.4)
	Radiation	28 (50.0)
	Chemoradiation	9 (16.1)
	Unknown	2 (3.6)

AJCC American Joint Committee on Cancer, ENE extranodal extension, TL total laryngectomy

^a^Acc. to the 8th edition of the AJCC Cancer Staging Manual 2017

Neck dissection investigation showed a mean NY count of 56.6 ± 27.27 with a mean LNB of 1.11 ± 1.82 resulting in a mean LNR of 0.03 ± 0.10. Calculation of LODDS resulted in a range from − 2.48 to 0.37 with a mean value of − 1.68 ± 0.50 (Table 2).

**Table 2:** Lymph node count features

	Min. – Max	Mean (± SD)	Median
NY	4–149	56.6 ± 27.27	54
LNB	0–7	1.11 ± 1.82	0.00
LNR	0.00–0.75	0.03 ± 0.10	0.00
LODDS	-2.48 – 0.37	-1.68 ± 0.50	-1.79

NY nodal yield, LNB lymph node burden, LNR lymph node ratio, LODDS log odds of positive lymph nodes, SD standard deviation, LNR lymph node ratio, LODDS log odds of positive lymph nodes

**Fig. 1:**
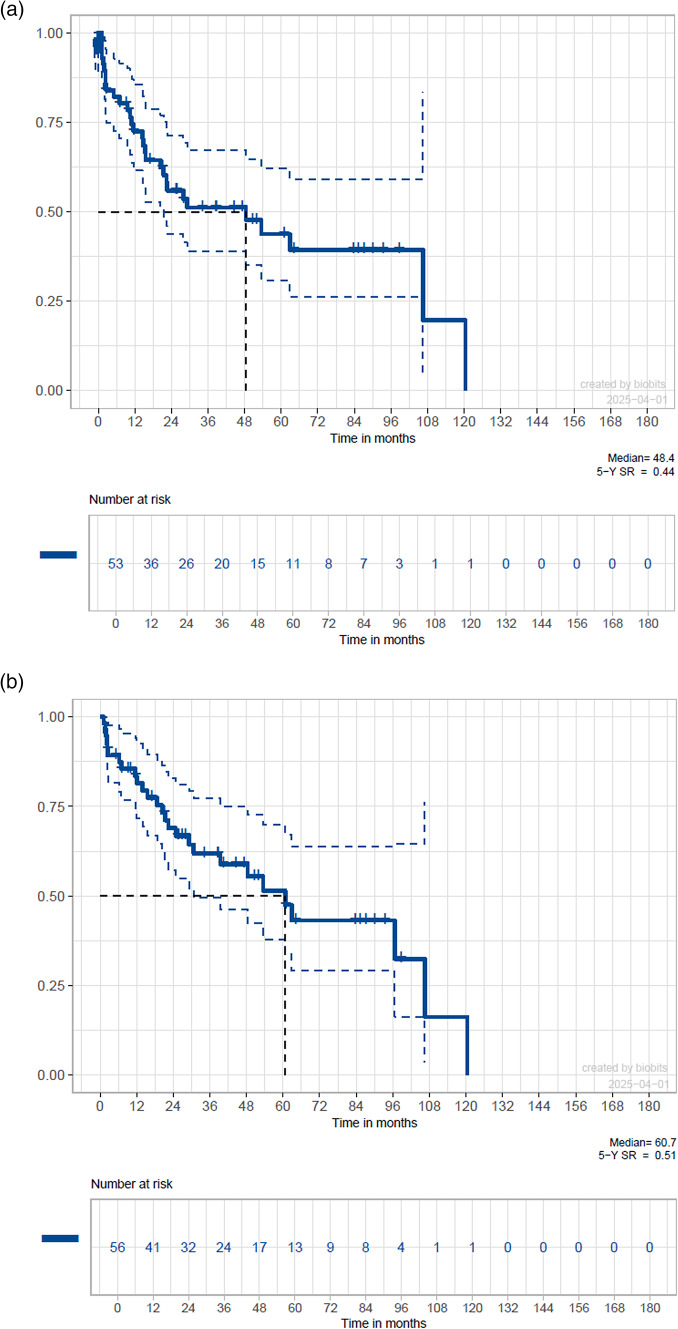
Kaplan–Meier curves for estimated survival probability of the whole study cohort. A 5-year disease-free survival of 44% and median disease-free survival of 48.4 months becomes evident (**a**). A 5-year overall survival of 51% and median overall survival of 60.7 months is displayed (**b**)

Survival analysis for the whole cohort revealed a 5-year DFS of 44% and median DFS of 48.4 months (Fig. 1a), while a 5-year OS of 51% and median OS of 60.7 months was estimated (Fig. 1b).

**Fig. 2:**
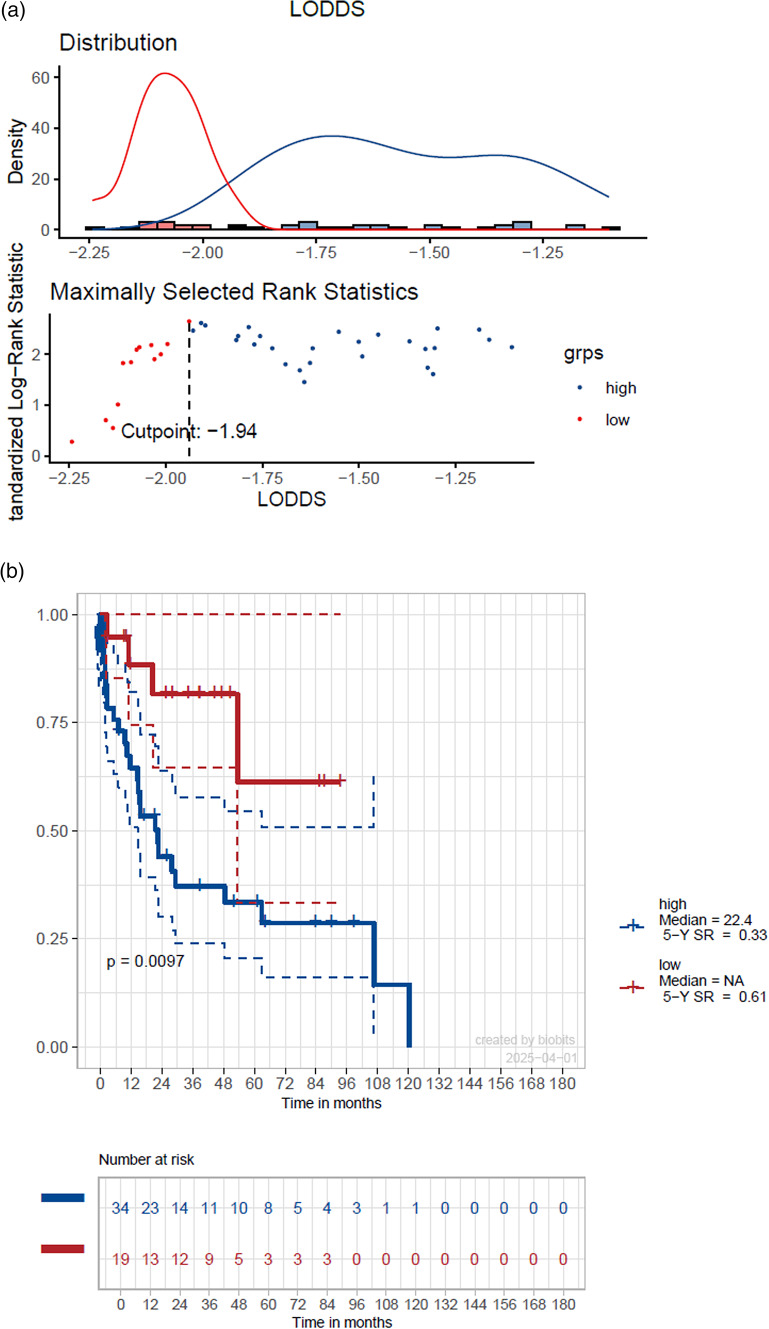

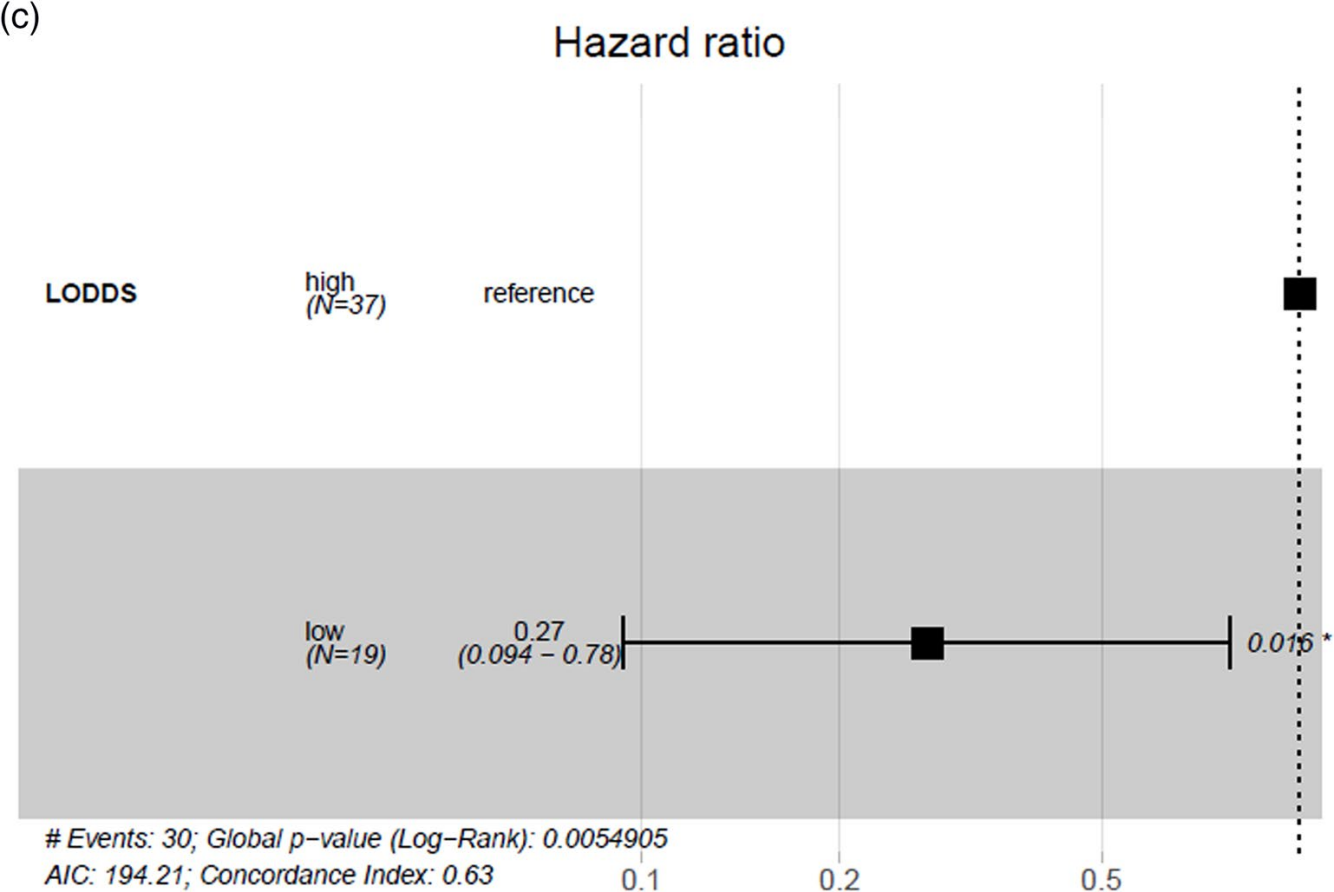
Estimated survival using LODDS stratification with an optimized cut-off point value of –1.94 (**a**) showing a 5-year disease-free survival of 33% vs. 61% (**b**) with a hazard ratio of 0.27 (*p* = 0.005) (**c**)

When stratifying the cohort at the mean values of LNB, LNR and LODDS no significant differences in DFS were found (*p* > 0.05). After using optimized cut-off point calculation significant differences in DFS for all three parameters became evident (*p* < 0.05), showing the highest concordance index for LODDS of 0.63 (Table 3). The cut-off at –1.94 for LODDS showed a 5-year DSF of 33% vs. 61% with a HR of 0.27 (*p* = 0.005) (Fig. 2).

**Table 3:** Univariate regression analysis on DFS stratified on mean values, resp. optimized cut-off points

	Mean value stratification	5 year DFS (%)	Median DFS (months)	HR (95% CI)	Significance, (global p-value)	Concordance index	Optimized cut-off point stratification	5 year DFS (%)	Median DFS (months)	HR (95% CI)	Significance, (global p-value)	Concordance index
LNB	0 vs. ≤ 1.11 vs. > 1.11	58 vs. 43 vs. 20	62.8 vs. 29.2 vs. 21.4	0.83 (0.27 − 2.6) vs. 1.69 (0.53 − 5.3)	0.238	0.55	≤ 2 vs. > 2	52 vs. 11	62.8 vs. 15.4	0.41 (0.18 − 0.91)	**0.039**	0.57
LNR	0 vs. ≤ 0.03 vs. > 0.03	58 vs. 50 vs. 11	62.8 vs. 29.2 vs. 21.4	0.96 (0.31 − 3.0) vs. 2.19 (0.69 − 6.9)	0.138	0.56	≤ 0.04 vs. > 0.04	53 vs. 0	62.8 vs. 11.5	0.3 (0.13 − 0.7)	**0.011**	0.58
LODDS	≤ -1.68 vs. > -1.68	60 vs. 25	106.5 vs. 22.4	2.0 (0.93 − 4.2)	0.073	0.56	≤ -1.94 vs > -1.94	61 vs. 33	n.a. vs. 22.4	0.27 (0.094 − 0.78)	**0.005**	0.63

Significant values (p < 0.05) are marked bold

**Fig. 3:**
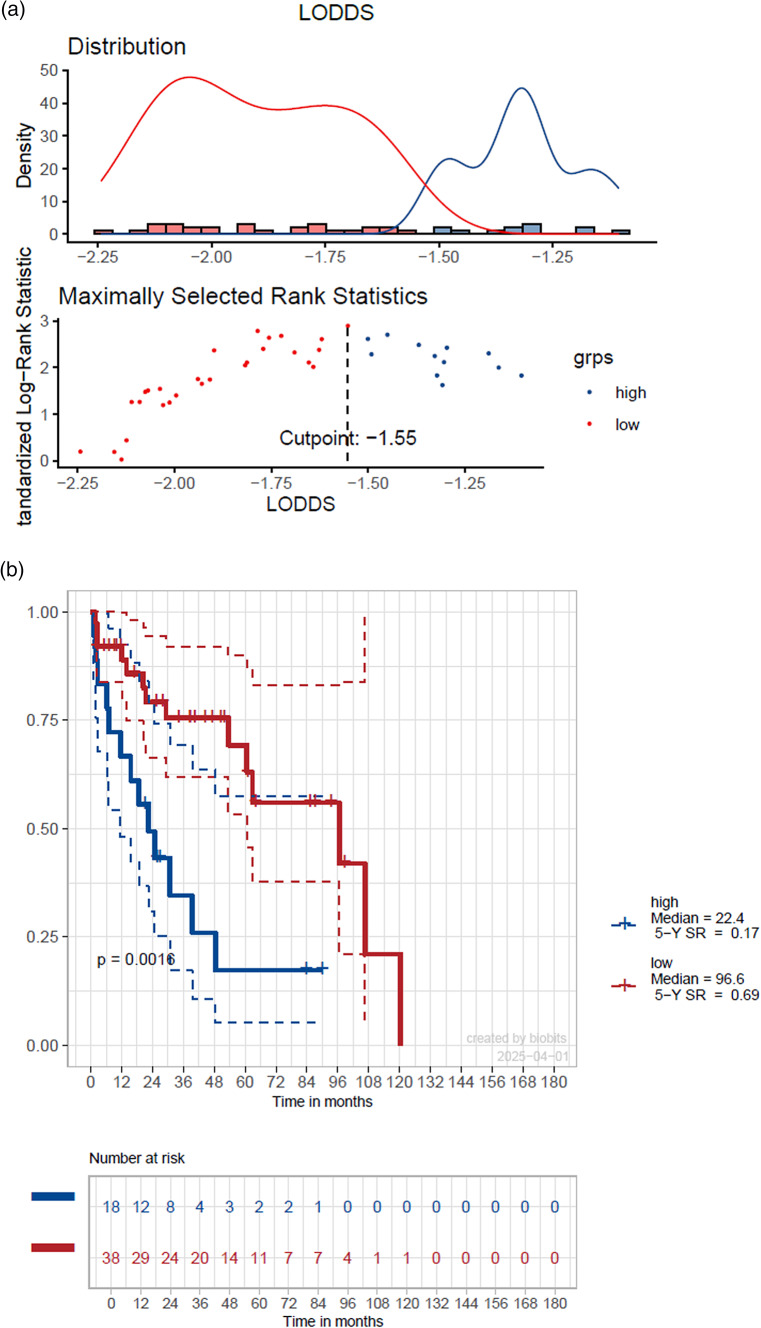

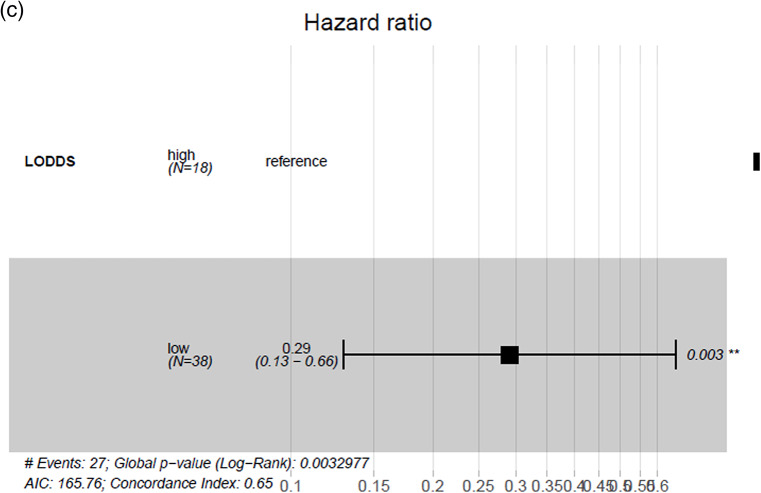
Estimated survival using LODDS stratification with an optimized cut-off point value of –1.55 (**a**) showing a 5-year overall survival of 17% vs. 69% (**b**) with a hazard ratio of 0.29 (*p* = 0.003) (**c**)

Estimation for OS revealed significant differences only for LODDS when stratified at its mean value (≤ -1.68 vs. > -1.68) showing a 5-year OS of 68 vs. 32 months (HR: 2.7, *p* = 0.019) while LNB and LNR showed no statistical significance (*p* > 0.05) (Table 4). The optimized cut-off of -1.55 showed significant differences in 5-year OS (69 vs. 17%, HR: 0.29, *p* = 0.003) and with a value of 0.65 demonstrating the highest concordance index of all investigated parameters (Fig. 3). LNB and LNR also showed significant OS differences when separated on optimized cut-off values of LNB: 2 and LNB: 0.04 (*p* < 0.05) but with lower significance and lower concordance indices (0.59 each) (Table 4).

**Table 4:** Univariate regression analysis on OS stratified on mean values, resp. optimized cut-off points

	Mean value stratification	5 year OS (%)	Median OS (months)	HR (95% CI)	Significance, (global p-value)	Concordance Index	Optimized cut-off point stratification	5 year OS (%)	Median OS (months)	HR (95% CI)	Significance, (global p-value)	Concordance Index
LNB	0 vs. ≤ 1.11 vs. > 1.11	66 vs. 43 vs. 31	96.6 vs. 30.7 vs. 39.4	1.34 (0.42 − 4.3) vs. 0.5 (0.17 − 1.8)	0.141	0.58	≤ 2 vs. > 2	62 vs. 15	96.6 vs. 22.4	0.36 (0.15 − 0.86)	**0.031**	0.59
LNR	0 vs. ≤ 0.03 vs. > 0.03	66 vs. 62 vs. 12	96.6 vs. 60.7 vs. 24.8	2.24 (0.69 − 7.3) vs. 0.74 (0.23 − 2.4)	0.059	0.59	≤ 0.04 vs. > 0.04	61 v. 0	96.6 vs. 18.7	0.27 (0.11 − 0.68)	**0.011**	0.59
LODDS	≤ -1.68 vs. > -1.68	68 vs. 32	96.6 vs. 30.7	2.7 (1.2 − 6.1)	**0.019**	0.61	≤ -1.55 vs. > -1.55	69 vs. 17	96.6 vs. 22.4	0.29 (0.13 − 0.66)	**0.003**	0.65

Significant values (p < 0.05) are marked bold

## Discussion

Our work indicates the necessity for improving survival rates in ALSCC since the oncologic outcome with a 5-year OS of 51% remains poor. This study was able to define cut-off values for risk stratification based on lymph node count features. These presented thresholds might help to define high-risk from low-risk patients with regard to OS and DFS in ALSCC undergoing curative intent TL with bilateral ND. Furthermore, we were able to show that LODDS have a greater prognostic value than LNB and LNR.

There are several large retrospective studies which investigate the prognostic role of NY, LNB, LNR and LODDS in HNSCC in general and oral cancer in particular (Kouka et al. 2022; Safi et al. 2017; Jin et al. 2020; Yildiz et al. 2016; Subramaniam et al. 2019; Roberts et al. 2016) However, there is marked divergence towards the relative robustness of the individual parameters. First, it is not clear whether parameters which incorporate the number of resected lymph nodes (such as LNR and LODDS) are at all superior to the sheer number positive nodes or the pN + status to evaluate prognosis: while many studies of reasonable size (several hundreds of patients each) advocate for compound parameters such as LNR and LODDS (Safi et al. 2017; Jin et al. 2020; Lee et al. 2017), a large database analysis of 12 437 patients by Roberts et al. showed that the number of positive nodes demonstrated superior prognostic value in comparison with the LNR and even the AJCC N staging (Roberts et al. 2016).

LODDS is the most complex lymph node parameter in this study: by adding 0,5 on each side of the quotient, LODDS can also compare patients with no positive nodes where LNR would be 0. Indeed, some studies also demonstrate the superiority of LODDS over the other parameters (Kouka et al. 2022; Safi et al. 2017; Jin et al. 2020; Yildiz et al. 2016). We aim to add this matter to the body of data by evaluating ALSCC originating foremost from the true vocal fold in particular, since it represents a distinct subgroup of HNSCC with a relatively low regional metastasis rate appearing mainly in advanced stages compared to other HNSCC entities like e.g. oropharyngeal cancer (Jansen et al. 2023).

In our cohort, a low number of positive nodes (mean LNB of 1.11 ± 1.82), a low value for LNR and a low value for LODDS were correlated with better OS, which is in line with most of the previous studies on that subject. Notably, the cut-off values for the positive impact on OS of both LNR and LODDS vary in the existing literature, which can be ascribed to varying statistical methods and heterogeneous patient populations (sample sizes and entities). Therefore, our data provide thresholds for one certain entity in advanced stage receiving one distinct therapy.

Even though being accepted as a quality marker for ND and provided with an ipsilateral minimum of n = 18 in general, NY impact on survival in ALSCC remains uncertain (Böttcher et al. 2016, 2021). Notably, Bao et al. reported in their study, which included 706 patients with oral cavity cancer, a complex relationship between NY and survival: the hazard ratio was reduced with each additional negative lymph node dissected up to 24, and increased when the number of negative lymph nodes was greater than 40 (Bao et al. 2020). Overall, these findings suggest that an extensive lymph node removal can also be detrimental to OS and should be carefully weighted against the expected reduction in tumor burden. Further work should be done to establish clinically relevant, entity-related, and comparable thresholds of LNB, LNR and LODDS that might help evaluate prognosis and the necessity for adjuvant therapy even in supposed low-risk cases. LODDS might serve as a risk feature helping to differentiate between ALSCC cases needing postoperative therapy escalation and those that do not (de-escalation).

The limitations of this study include its limited sample size, the retrospective nature, the lack of histological work-up guidelines for ND specimen, and the surgeons’ arbitrary approaches in ND, at least in cases before 2011, after which time a consistent surgical technique was performed in our institution on a regular basis (Lörincz et al. 2016). The cut-off estimation for the lymph node count features is subject to a data-driven cut point bias as it was determined on an exploratory basis. Its necessity lied in the limited sample size on which a cut-off calculation resulted in unreasonable figures. Additionally, postoperative (adjuvant) treatment plans and individual adherence to it might have a bigger impact on oncological outcomes. A prospective validation of our results in a randomized controlled manner including multiple centers is therefore recommended. The strengths of this study are foremost its restrictively condensed cohort of only TL cases with a small corridor of clinical features.

## Conclusion

We propose LODDS to serve as a superior prognosticator compared to LNB and LNR concerning DFS and OS in TL for ALSCC. LODDS values of -1.94 for DFS and -1.55 for OS appear to be suitable thresholds for risk stratification. A prospective validation of our results is needed.

## Data Availability

No datasets were generated or analysed during the current study.
